# Psychometric Properties of the Farsi Version of Eyberg Child Behavior Inventory (F-ECBI) in Iranian Population

**DOI:** 10.18502/ijps.v15i4.4300

**Published:** 2020-10

**Authors:** Samiyeh Panahandeh, Hamid Poursharifi, Behrooz Dolatshahi, Asma Aghebati

**Affiliations:** 1 Department of Clinical Psychology, University of Social Welfare and Rehabilitation Sciences, Tehran, Iran.; 2 Department of Clinical Psychology, School of Behavioral Sciences and Mental Health (Tehran Institute of Psychiatry), Iran University of Medical Sciences, Tehran, Iran.

**Keywords:** *Behavior Problems*, *Children*, *Eyberg Child Behavior Inventory*, *Iran*, *Psychometric Properties*, *Parent Reports*

## Abstract

**Objective:** Eyberg Child Behavior Inventory (ECBI) is one of the most frequently used tools for measuring behavioral problems; however, no research has been done to evaluate its psychometric properties in Iran.

**Method** **:** The present study sought for exploring the factor structure and psychometric properties of the F-ECBI in an Iranian sample. A total of 495 mothers (mean age = 33.83 years; SD = 4.78) who reported behavioral problems in their children aged 3 to 12 years (mean age = 6.36 years; SD = 2.66) were selected via convenience sampling in 2018-2019. The psychometric properties of F-ECBI, including reliability (Cronbach’s alpha) and validity (exploratory and confirmatory factor analysis, and convergent validity) were assessed using SPSS version 25 and LISREL 8.80.

**Results: **By performing EFA on the first sample part (n = 360), the examination of scree plot supported a 3-factor or 4-factor solution, and pattern matrix resulted in a 3-factor structure. The factors were called as “behavioral problems related to oppositional defiant”, “behavioral problems related to inattentive”, and “behavioral problems related to conduct”, according to their content and the research. CFA was performed on the second part of the sample (n = 135) to test the fitness of the 3-factor solution. According to CFI (0.91), NFI (0.91), NNFI (0.90), IFI (0.91), PNFI (0.77), GFI (0.75) AGFI (0.70), PGFI (0.62) and chi-square (540.31) indexes, the model had acceptable fitness. Cronbach's alpha was employed to measure the internal consistency and it revealed to be at a good to excellent level (behavioral problems related to oppositional defiant = 0.88; behavioral problems related to inattentive = 0.84; behavioral problems related to conduct = 0.74). The 3-factors subscales were associated with total difficulties, internalizing and externalizing SDQ, indicating the good convergent validity of F-ECBI.

**Conclusion: **The F-ECBI has good psychometric properties in Iran and can be employed as a useful instrument for measuring children's behavioral problems.

Psychological, emotional, and behavioral disorders at a young age are a serious concern in the field of public health (1-3). The suffer the patient and his family experience, limited ability to achieve the common goals of academic and social life, performing below the optimal level, and imposing heavy costs on the community due to the need for care all convey the high importance of these disorders (2). Prevention of mental, emotional, and behavioral disorders at an early age is an effective investment (2, 4, 5). Among the advantages are prevention, lower costs of treatment, less suffering, fewer premature mortality, a happier family, and more successful people. 

As a result, practical action in this area will lead to these benefits (2). 

High levels of behavioral problems in children are a serious risk factor with negative outcomes at a older ages (6, 7). Some examples related to behavioral problems at an early age are crime, violence, and fight (8-10), substance and alcohol abuse (8, 11), subsequent mental disorders (12, 13), problems in academic performance (14-17), job and employment problems (18), increased mortality (19), and poor social and intimate relationships (20).

Despite the prevalence of behavioral and emotional problems during childhood and its significant contribution to future problems, there is a potential for lack of diagnosis and timely treatment (21).

Behavioral measurements for screening these behavioral problems in children and more quickly identifying those children with problems for referring them to interventions and treatments can be useful. There is a large number of instruments for identifying children's behavioral problems, but only a few have acceptable psychometric properties (3). One of the most widely used and valid measurements of behavioral problems is the Eyberg Child Behavior Inventory (ECBI), created by Eyberg and Pincus (22) to track these difficulties in 2-16 year-old children (22). This questionnaire contains 36 items on behavioral problems that are filled out by parents on 2 scales of “intensity” and “problem.” The scale of intensity measures how often a certain behavior occurs on a Likert scale ranging from “never” to “always”, and the parent can response with a “Yes” or “No” on the problem scale to report if this behavior is problematic to his/her or not.

Parents' reports on children's behavioral problems can be highly important due to their observation in a variety of situations over a long period of time, making them one of the most important sources for assessing children's emotional-behavioral symptoms (23). There are several measurements for evaluating behavioral problems in Iran and the most commonly used are the Child Behavior Checklist (CBCL), the Strengths and Difficulties Questionnaire (SDQ), and the Conner’s Comprehensive Behavior Rating Scale (CBRS). Being lengthy and taking considerable time to respond, low age range, difficulty in administration and scoring, having several separate forms for different age groups, and not being specific to behavioral problems are some of the limitations of some behavioral problem evaluation questionnaires. Shortness, fast administration and scoring, simple interpretation, and usability in different contexts are the distinguishing features of ECBI compared with other tools, such as CBCL and SDQ, which has made it a widely used measurement (24). In early diagnosis and intervention, early screening of the child's behavioral problems is essential, and ECBI is one of the best tools for primary screening of symptoms. The tool is also able to monitor and detect behavioral changes over time and provide information about achieving therapeutic goals, which make it a suitable tool for use in therapeutic interventions (25).

There are several translations of ECBI in different languages and it has been employed in various countries. The validity of this questionnaire have been examined in countries such as the United States (26-28), the Netherlands (3, 24, 29), Norway (30, 31), Sweden (3), Finland (32), Australia (33), South Korea (34), Spain (35) and Taiwan (36). In examining the psychometric properties and factorial structure of this questionnaire in different cultures, different results have been obtained. In the Netherlands (24) and Finland (32), for example, they have achieved a one-dimensional structure by conducting exploratory factor analysis (EFA), as the original version of the questionnaire suggests. However, Burns and Patterson (26, 27) achieved a 3-factor structure in the United States, and several studies were designed and implemented to test this 3-factor model (3, 28, 30), by using EFA and CFA which confirmed their 3-factor model. Moreover, an 8-factor structure was found in South Korea (34) and a 2-factor in Australia (33). Cronbach's alpha values showed high values representing good internal consistency of the measurement in Dutch (24), Swedish (3), Finish (32), South Korean (34), and Norwegian (31) samples, ranging from 0.91 to 0.95, except for Spanish (35) sample with the Cronbach's alpha of 0.73. However, no research in Iran has been done so far to test the validity and reliability of this questionnaire. Therefore, the goal of this study was exploring the psychometric properties of ECBI in Farsi.

## Materials and Methods

***Participants***

We tried to assess the psychometric properties of the ECBI in the Iranian children aged 3-12 years old (based on mother's report) and the children at this age residing in Tehran were the target population of this cross sectional study. Considering the cultural-geographical distribution and different regions of Tehran, 495 participants from kindergartens and schools in Tehran were selected by convenience sampling method in 2018-2019. Districts of 2, 5, 4, 8, 11, 12, 17, and 18 were considered and from each district a kindergarten and a school were selected for sampling. The entry criteria for this research were having a child aged between 3 and 12 years and willingness to participate in the study. 

***Procedure***

The ECBI was translated into Farsi; then, it was back translated to English by 2 independent translators. Subsequently, an expert panel consisting 3 psychologists, discussed their views on the questionnaire and made some changes. In the next step for initial evaluation, this version of the questionnaire was administered to 20 mothers who had children aged 3 to 12 years and their views on the items were considered. At the beginning of the study, the participants were informed about the privacy and confidentiality regulations, voluntarily participation, research objectives, research field, provision of the results if desired, the average time needed to complete the measurement, and its application for only research purposes. Participants filled out the ECBI, Strengths and Difficulties Questionnaire (SDQ), and informed consent in written form.

***Ethics***

The study achieved the approval of the ethics committee of the University of Social Welfare and Rehabilitation Sciences of Tehran with approval number of IR.USWR.REC.1397.075 on 24.09.2018.

***Statistical Analyses***

Children’s age ranged from 3 to 12 (mean age = 6.36 years; SD = 2.66). The participants showed the following proportions: 3 years old (16.6%), 4 years old (15.2%), 5 years old (12.1%), 6 years old (13.1%), 7 years old (10.7%), 8 years old (9.3%), 9 years old (6.7%), 10 years old (7.1%), 11 years old (5.3%), and 12 years old (4%). Also, 41.2% of the children were girls and 58.8% were boys; 51.1% were the only child, 37% were the first child, 2% were the middle child, and 9.9% were last child. Mothers with an age range of 21 to 56 participated (mean age = 33.83 years; SD = 4.78). Of them, 71.9% were housewives and 28.1% employed; 3.8% were undergraduates, 22.4% had high school diploma, 11.9% associate degree, 42.8% bachelor’s degree, 16.4% master’s holder, and 2.6% PhD and above. 

No missing data protocols were used for EFA (ie, 1:10; (37)) because no data were missing for F-ECBI for the first sample part of the participants (ie, n = 360). Bartlett’s tests of sphericity and the Kaiser-Meyer-Olkin measure of sampling adequacy were employed to check the factor structure of the ECBI items indicating 0.60 or higher values were appropriate for factor analysis (38). EFA with principal axis and promax rotation was performed to test the factor structure of the F-ECBI. The scree plot determined the number of factors (39, 40), an examination of the Kaiser-Guttman criterion (41, 42). Minimal factor loadings were set to 0.32, per recommendations (43, 44). For the second part of the participants (n = 135), confirmatory factor analysis (CFA) was used to test the fit of the standard F-ECBI structure (45). RMSEA values were less than about 0.08 or below, the CFI, values of about >0.95, with acceptable fit >0.90 (46). Moreover, internal consistency of the F-ECBI was calculated using Cronbach’s alpha coefficients. Internal consistency standard is α ≥ 0.70 (47, 48). Furthermore, with respect to convergent validity, we used Pearson correlating (PCC) to examine the relationship between the F-ECBI subscales with SDQ subscales. Accordingly, in terms of the general guidelines for the magnitude of correlations in behavioral sciences (49), r values equal to 0.10, 0.30, and 0.50 could be viewed as small, moderate, and large correlations, in turn. We used Skewness and Kurtosis to examine the normality of the F-ECBI scores. Analyses were conducted using SPSS v.25, except for the CFA, which were conducted using LISREL version 8.80. Statistical significance level was at p < 0.05; all tests were 2-tailed.

## Results

**Figure 1:**
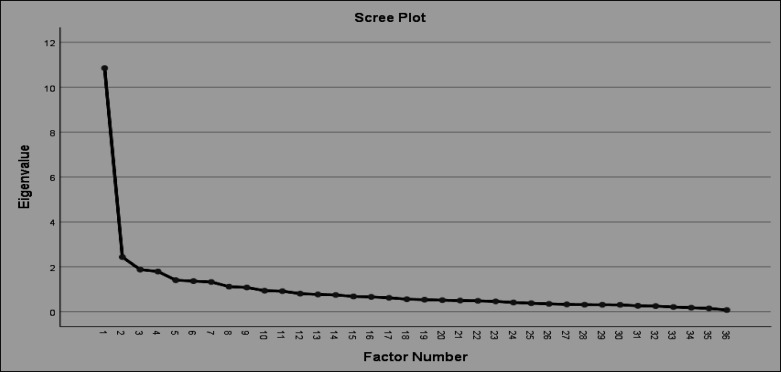
Scree Plot of the Farsi Version of Eyberg Child Behavior Inventory (F-ECBI) Factors

**Figure 2:**
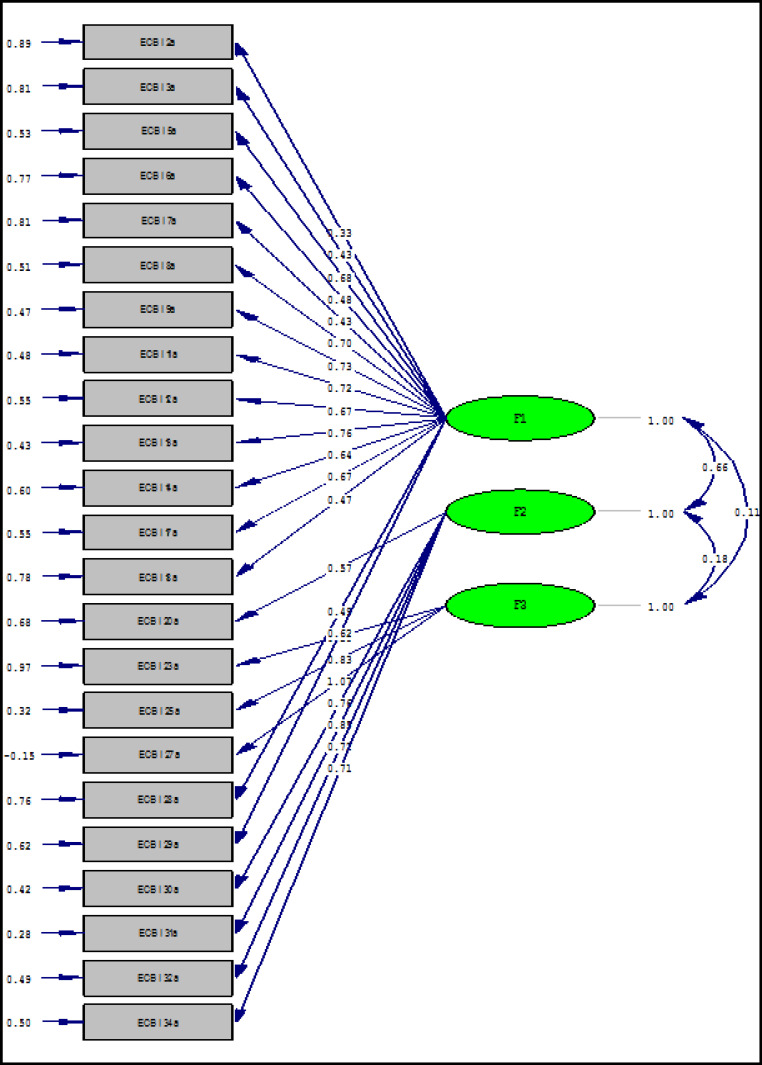
Factor Loading of the Farsi Version of Eyberg Child Behavior Inventory (F-ECBI) Factors in Confirmatory Factor Analysis (CFA)

*Exploratory Factor Analysis.* EFA using the principal axis factoring with promax rotation indicated that the F-ECBI items were appropriate for factor analyses for participants (Table 1). Bartlett’s test of sphericity was significant (χ2 = 6427.022, df = 630, p < 0.001) and the Kaiser-Meyer-Olkin was 0.89. Additionally, examination of eigenvalues and a 3-factor solution was proposed by scree plot (Figure 1). The pattern matrix indicated that the items loaded strongly onto 3 factors (loading of 0.40 or higher). The items in factor 1 (ie, behavioral problems related to oppositional defiant; 2, 3, 5, 6, 7, 8, 9, 11, 12, 13, 14, 17, 18, 28, 29), Factor 2 (ie, behavioral problems related to inattentive; 20, 30, 31, 32, 34), and factor 3 (ie, behavioral problems related to conduct; 23, 25, 27) were reported (Figure 2). Furthermore, the other items had cross loading or low factor loading.

*Confirmatory Factor Analysis.* Although RMSEA (ie, 0.09) was higher than 0.08, the 3-factor solution altogether using CFA provided relatively acceptable to the data (CFI = 0.91, NFI = 0.91, NNFI = 0.90, IFI = 0.91, PNFI = 0.77, GFI = 0.75, AGFI = 0.70, PGFI = 0.62, chi-Square (df) = 540.31 (227), p < 0.0001).

Internal Consistency and Intercorrelations. Cronbach’s alpha values ranged from 0.74 (Factor 3; Behavioral problems related to conduct) to 0.88 (Factor 1; Behavioral problems related to oppositional defiant) exhibiting good to excellent internal consistency of the F-ECBI. Furthermore, all correlations among the F-ECBI subscales were small to medium. Further, the data of F-ECBI scores were normal as Skewness and Kurtosis were between ±2 (Table 2 for more information).

*Convergent Validity.* The Pearson correlation (PCC) indicated that the F-ECBI subscales were positively associated with total difficulties, and internalizing and externalizing SDQ scales (Table 3).

**Table 1:** Exploratory Factor Analysis of the Farsi Version of Eyberg Child Behavior Inventory (F-ECBI) Items

**Item**	**Factor 1** **Oppositional defiant**	**Factor 2** **Inattentive**	**Factor 3** **Conduct**
13 Has temper tantrums	0.765	-0.088	0.148
12 Get angry when doesn’t get own way	0.758	-0.047	0.017
7 Refuses to go to bed on time	0.702	-0.079	-0.145
6 Slow in getting ready for bed	0.696	-0.058	-0.159
17 Yells or screams	0.673	-0.146	0.266
18 Hits parents	0.584	-0.068	0.021
3 Has poor table manners	0.581	-0.030	-0.029
14 Sasses adults	0.543	-0.086	0.225
2 Dawdles or lingers at mealtime	0.527	-0.084	-0.226
11 Argues with parents about rules	0.492	0.186	0.100
5 Refuses to do chores when asked	0.476	0.215	0.094
9 Refuses to obey until threatened with punishment	0.468	0.184	0.164
8 Does not obey house rules on own	0.424	0.230	0.161
29 Interrupts	0.423	0.269	-0.132
28 Constantly seeks attention	0.418	0.187	0.018
34 Has difficulty concentrating on one thing	-0.206	0.912	0.021
31 Has short attention span	-0.161	0.890	0.033
30 Is easily distracted	-0.072	0.810	-0.009
32 Fails to finish tasks or projects	0.097	0.643	-0.012
20 Is careless with toys and other objects	-0.015	0.567	0.021
25 Verbally fights with sisters and brothers	-0.204	-0.084	0.869
27 Physically fights with sisters and brothers	-0.210	-0.025	0.855
23 Teases or provokes other children	0.129	0.191	0.416
1 Dawdles in getting dressed	0.422	0.200	-0.351
10 Acts defiant when told to do something	0.440	0.357	-0.020
4 Refuses to eat food presented	0.379	-0.024	-0.044
35 Is overactive or restless	0.374	0.218	0.006
16 Cries easily	0.357	0.018	0.012
15 whines	0.334	0.230	-0.005
19 Destroys toys and other objects	0.072	0.371	0.062
22 Lies	-0.020	0.361	0.252
21 Steals	0.044	0.117	0.222
24 Verbally fights with friends own age	0.042	0.222	0.386
26 Physically fights with friends own age	0.116	0.168	0.339
33 Has difficulty entertaining self alone	0.222	0.396	-0.162
36 Wets the bed	0.094	0.108	-0.012
Eigenvalues	10.28	1.85	1.34
% Explained variance	28.56	5.16	3.75

Factor loading and eigenvalues obtained using principal axis factoring with promax rotation. Factor loading ≥0.40 in boldface. Cross loading and low factor loading in Italic.

**Table 2:** Internal Consistency, Descriptive, Correlations, Skewness and Kurtosis among Farsi Version of Eyberg Child Behavior Inventory (F-ECBI) Subscales

**ECBI Subscales**	**Internal Consistency**	**Means (SD)**	**1**	**2**	**3**
1. Factor 1 Behavioral problems related to oppositional defiant	0.88	48.17 (16.18)			
2. Factor 2 Behavioral problems related to inattentive	0.84	13.60 (7.08)	0.61***		
3. Factor 3 Behavioral problems related to conduct	0.74	5.11 (4.38)	0.25**	0.24**	
Skewness			0.755	0.992	1.099
Kurtosis			0.235	0.286	0.398

*** p<0.001,

** p<0.01

**Table 3:** Pearson Correlations between Farsi Version of Eyberg Child Behavior Inventory (F-ECBI) and Strengths and Difficulties Questionnaire (SDQ) Subscales

	**Total difficulties** 1	**Externalizing**	**Internalizing**
Factor 1. Behavioral problems related to oppositional defiant	0.67	0.63	0.54
Factor 2. Behavioral problems related to inattentive	0.69	0.70	0.47
Factor 3. Behavioral problems related to conduct	0.38	0.42	0.21

Note: All correlations are significant at p<0.0001.

1 . Summed externalizing and internalizing scores.

## Discussion

The present work was carried out to evaluate the psychometric properties and factor structure of the Farsi version the Eyberg Child Behavior Inventory (F-ECBI). By performing EFA on the first sample part (n = 360), the pattern matrix of scree plot indicated that the items loaded strongly on 3 meaningful factors. The factors were named “behavioral problems related to oppositional defiant”, “behavioral problems related to inattentive”, and “behavioral problems related to conduct”, according to their content and the research. CFA was performed on the second part of sample (n = 135) to test the fitness of 3-factor solution. According to CFI, NFI, NNFI, RMSEA, and chi-Square indexes, CFA had acceptable fitness. To assess internal consistency, Cronbach’s alpha was calculated and the results indicated good to excellent values. Three factors of F-ECBI were associated with total difficulties, internalizing and externalizing SDQ subscales that indicated good convergent validity.

The analyses confirmed convergent validity and internal consistency for this instrument. These results were approved the findings of other researchers in other countries such as Norway (31), Sweden (3), Korea (34), Australia (33), Taiwan (36), and the Netherlands (24). The results of EFA indicated 3 significant factors (behavioral problems related to oppositional defiant, behavioral problems related to inattentive, and behavioral problems related to conduct), which were from a clinical viewpoint meaningful factors. In the CFA, these 3 factors were confirmed. The developer of this measure has considered its intensity subscale to be unidimensional (22); however, there are disagreements and various studies have achieved different results. Some studies have backed the unidimensional structure of this inventory (24, 32, 50, 51), while others found more than one factor in its structure (3, 26-28, 30, 33, 34). Having subscales for identifying behavioral problems in children enables us to select more homogenous sample of children with similar symptoms in each subscale. Another problem could be that if our aim is to choose the children for parenting interventions with high levels of oppositional defiant behavior, a higher score on behavioral problems related to oppositional defiant subscale is better than high scores in ECBI. Besides, behavioral problems related to conduct can be considered as severity of the problem. Compared to having just one total score, subscales would be more sensitive to interventions and can indicate changes on different domains of problems (27). In general, considering the 3-factor structure of this instrument, having 3 separate conceptual subscales can be more useful for diagnosis and comparison in research and intervention, compared to a general score of behavioral problems.

## Limitations

Despite obtaining good reliability and validity of the F-ECBI in the present study, the current work also had some limitations. For example, since a part of the sample was not accessible for retesting, conducting a test-retest reliability was not possible in this study. Also, a high percentage of the sample was between the ages of 3 and 8, which requires further research on older children to achieve acceptable generalizability of the results. The assessment of the psychometric properties of the measurement in the clinical population and its comparison with the results of nonclinical population in further research can also be beneficial. This study was conducted on the sample of Tehran by using convenient sampling method, which is not representative of general population. Replication of this study in larger samples in Iran is also recommended.

## Conclusion

The F-ECBI has good reliability and validity for use in Iran. Such research allows accessing instruments for measuring children's problems in cultural context. Besides, in reviewing the factor structure of this inventory, 3 subscales were explored, while the original version is unidimensional.
